# Epiglottis Cartilage, Costal Cartilage, and Intervertebral Disc Cartilage as Alternative Materials in the Postmortem Diagnosis of Methanol Poisoning

**DOI:** 10.3390/toxics11020152

**Published:** 2023-02-05

**Authors:** Marcin Tomsia, Elżbieta Chełmecka, Małgorzata Głaz, Joanna Nowicka

**Affiliations:** 1 Department of Forensic Medicine and Forensic Toxicology, Medical University of Silesia, 18 Medyków Street, 40-752 Katowice, Poland; 2 Department of Statistics, Department of Instrumental Analysis, Faculty of Pharmaceutical Sciences, Medical University of Silesia, Ostrogórska 30 Street, 41-200 Sosnowiec, Poland

**Keywords:** costal cartilage, epiglottis cartilage, fatal intoxication, formic acid, industrial alcohol, intervertebral disc cartilage, methanol poisoning, postmortem diagnosis

## Abstract

Alternative materials for postmortem diagnosis in the case of fatal poisonings are much needed when standard materials, such as blood and urine, are unavailable. The study presents a case of fatal mass methanol intoxication resulting from industrial alcohol consumption. The study aimed to determine methanol and formic acid concentrations in epiglottis cartilage, costal cartilage, and intervertebral disc cartilage and to analyze the correlation between their concentrations in cartilage tissues and the femoral blood. Methanol and formic acid concentrations in samples collected from 17 individuals (*n* = 17) were estimated using gas chromatography with flame ionization detection (GC-FID). Methanol concentration in the costal cartilage correlated with its concentration in the femoral blood (r = 0.871). Similar correlations were found for epiglottis cartilage (r = 0.822) and intervertebral disc cartilage (r = 0.892). Formic acid concentration in the blood correlated only with its concentration in urine (r = 0.784) and the epiglottis (r = 0.538). Cartilage tissue could serve as an alternative material for methanol analyses in postmortem studies. Formic acid, a methanol metabolite, does not meet the requirements for its presence determination in cartilage tissues.

## 1. Introduction

In forensic autopsies, blood and urine—classical matrices in forensic toxicology—can be degraded or potentially affected by postmortem redistribution, hence, they are not always available [1]. Therefore, alternative sampling materials are needed. In forensic toxicology, the list of alternative matrices includes oral fluid [2,3], hair [4,5], sweat [6,7], meconium [8,9], breast milk [10,11], vitreous humor [12,13], bile [14,15], and even insects [16,17]. However, the alternative materials present limitations, such as limited xenobiotic accumulation (according to physical–chemical properties), the eventual need for more sensitive analyses, or the inability to correlate xenobiotic concentrations with effects [18].

Cartilage is one of the matrices studied in the context of xenobiotic distribution. The cartilage morphotic elements are embedded in the extracellular matrix, composed of structural elements, such as collagen fibers that protect cellular DNA against environmental factors and proteoglycans that bind water. Both elements ensure cartilage flexibility [19]. Due to these properties, forensic scientists’ interest in cartilage tissues increasingly grows [20]. In forensic genetics, costal cartilage can serve as a DNA source in cases of individual identification [21], and fibrous tissue of the intervertebral disc allows for rapid genetic identification [22,23]. Unfortunately, cartilage hydration affects the ability to determine the levels of water-soluble xenobiotics [19]. However, costal cartilage has been successfully used to detect nitrite ions in fatal sodium nitrite poisoning [24]. Additionally, costal cartilage, ethanol [25], and isopropanol [26] concentrations positively correlated with their concentrations in the blood.

Methanol ingestion and consecutive poisoning is a rising problem, closely associated with high morbidity and mortality [27]. Alcohol dehydrogenase oxidizes methanol to formaldehyde, and subsequently, aldehyde dehydrogenase oxidizes formaldehyde to formic acid, which accounts for the associated anion gap metabolic acidosis and end-organ damage [28]. Pure methanol’s lethal dose ranges from 300 to 1000 mg/kg [29]. Methanol ingestion is usually fatal.

Methanol distribution in different tissues and body fluids after absorption is poorly understood. Our research focused on methanol and formic acid distribution in cartilage tissues sampled from 17 fatal victims of a mass intoxication with industrial alcohol who died between April and June 2022 in the Silesia Region (Poland) [30].

## 2. Materials and Methods

**Figure 1:**
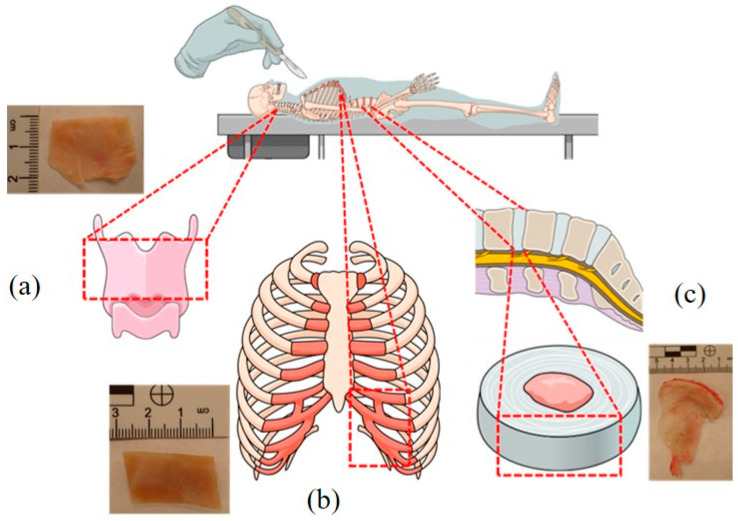
Anatomical location of the epiglottis (**a**), costal cartilage (**b**), and fibro-cartilage of the intervertebral disc (**c**). The dotted lines indicate the places of material sampling for testing. The diagram was prepared using Mind the Graph software (https://mindthegraph.com/ (accessed on 4 October 2022).

The samples were collected from 17 individuals who died due to methanol poisoning between April and June 2022 in the Silesia Region (Poland). The sample collection was approved by the Bioethical Commission of the Medical University of Silesia in Katowice (decision no. PCN/CBN/0052/KB/77/22, date of approval: 5 May 2022). Femoral blood, urine, costal cartilage, epiglottis, and fibro-cartilage of the intervertebral disc samples (Figure 1) were collected during medical–legal autopsies commissioned by the Prosecutor’s Office. All analyses were carried out in a certified forensic laboratory immediately after the autopsy.

The collected samples, 1 mL of fluid or 1 g of chopped cartilage tissue (devoid of soft tissues—thoroughly cleaned using three sterile scalpels, changed each time after removing the next surface layer, and then they were fragmented into 0.1 × 0.1 × 0.1 cm fragments), and then they were placed in 20 mL glass gas-tight vials, and analyzed as described by Tomsia et al. [25]. An eight-point calibration curve for methanol in mg/mL or mg/g (0; 0.1; 0.2; 0.5; 0.8; 1; 2; 3) was linear in the whole range. The limit of detection (LOD) was determined as 0.05 mg/mL or mg/g, and the limit of quantification (LOQ) was determined as 0.1 mg/mL or mg/g for the entire tested material. Linearity was maintained up to 5000 mg/L (R^2^ = 0.996).

Formic acid concentration in blood, urine, and tissues was determined using gas chromatography and the method described by Kuo et al. [31] and Abolin et al. [32]. In this method, formic acid was determined in the form of a volatile methyl formate ester. Using the FID detector (Thermo Fisher Scientific Inc., Milan, Italy) ensured the sensitivity of 0.01 mg/mL and reduced the impact of the biological background. The calibration curve for formic acid ranged from 0.1–2.0 mg/mL or mg/g.

The distribution of variables was evaluated using the Shapiro–Wilk test and the quantile–quantile plot. The interval data were expressed as mean values ± standard deviations. The regression analysis was used to determine the relationship between quantitative features. The data with non-normal distribution were log transformed before analysis. Comparisons of the ratios for blood/urine and blood/cartilage alcohol concentration between current and previous results [25] were made using the non-parametric U Mann-Whitney and Kruskal-Wallis tests, respectively. Statistical significance was set at a *p* < 0.05, and all tests were two-tailed. Statistical analysis was performed using Statistica, version 13.3 (TIBCO Software Inc., Palo Alto, CA, USA, 2017).

## 3. Results

The study group consisted of three women (18%) and 14 men (82%). Out of 17 victims, only four individuals (18%) were hospitalized. In five cases (30%), methanol in cartilage tissues was not detected. For these cases, the time from death to autopsy (t2) was 8 ± 5 (2–16) days (mean ± SD (min − max). The basic characteristics of quantitative variables are presented in the Table 1.

**Figure 2:**
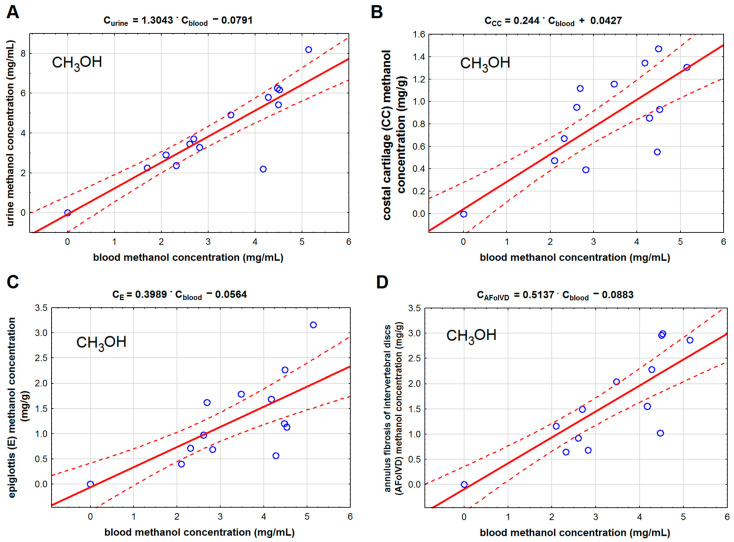
The ordinary least square regression model for the relationship between methanol (CH_3_OH) concentration in the blood and in: urine (**A**), costal cartilage (**B**), epiglottis (**C**), and anulus fibrosis of intervertebral discs (**D**). Legend: AFoIVD—annulus fibrosis of intervertebral discs, CC—costal cartilage, and E—epiglottis. The solid red lines represent regression lines, and the dashed lines indicate 95% confidence intervals.

**Figure 3:**
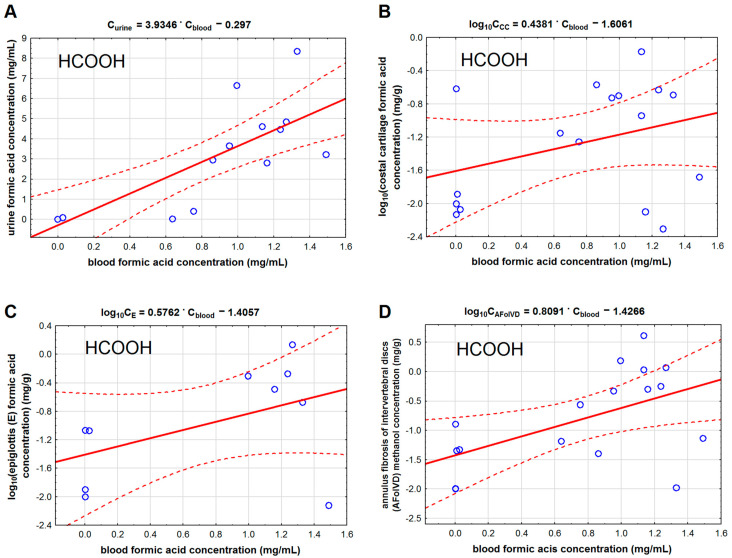
The ordinary least square regression model for the relationship between formic acid (HCOOH) concentration in the blood and in: urine (**A**), costal cartilage (**B**), epiglottis (**C**), and anulus fibrosis of intervertebral discs (**D**). Legend: AFoIVD—annulus fibrosis of intervertebral discs, CC—costal cartilage, and E—epiglottis. The solid red lines represent regression lines, and the dashed lines indicate 95% confidence intervals. The data for sections B, C, and D were log transformed before the analysis.

Methanol and formic acid concentrations in the studied cartilage tissues were much lower than in blood and urine. Among the studied cartilage tissues, the lowest concentrations of methanol and formic acid were found in the costal cartilage, and the highest were found in the annulus fibrosis of intervertebral discs. We found that methanol concentration in the costal cartilage (Table 2, Figure 2B) correlated with methanol concentration in the femoral blood (r = 0.871). We found the same correlation type for methanol concentration in the epiglottis (r = 0.822) and the fibro-cartilage of the intervertebral disc (r = 0.892). We also found that the formic acid concentration in the epiglottis cartilage (r = 0.538) correlated with its concentration in the blood (Figure 3).

Additionally, methanol and formic acid concentrations in the blood correlated with their concentrations in urine (r = 0.929 and r = 0.784, respectively).

### Alcohol Tissue Permeability Comparison

**Figure 4:**
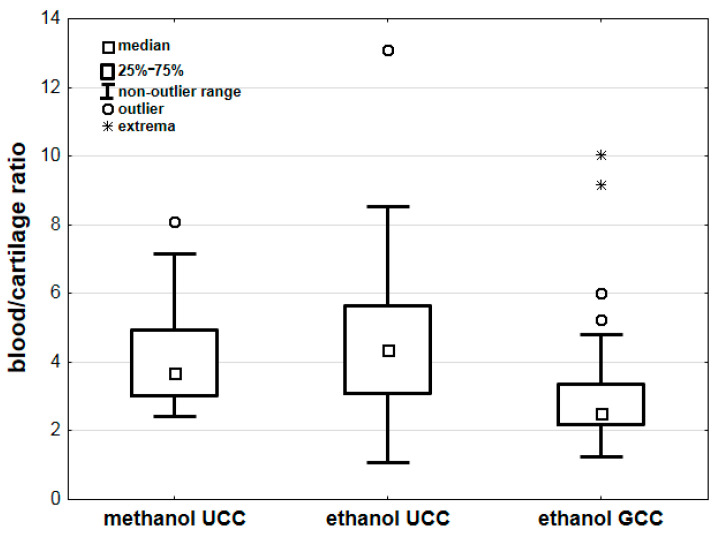
Comparison of blood/cartilage ratios for methanol and ethanol concentrations. Legend: GCC—costal cartilage prepared with the ground costal cartilage method [25], UCC—costal cartilage prepared with the unground costal cartilage method [25].

Comparing the results of methanol poisoning cases with cases of ethanol intoxication [25], we found non-statistically significant differences in blood/urine concentration ratios between methanol and ethanol (Table 3). However, we found statistically significant differences in the blood/cartilage concentration ratios for methanol and ethanol (*p* < 0.001; Table 3). Additional analyses showed no significant differences between the blood/cartilage ratios for methanol and ethanol (*p* = 1.000) concentrations determined using the UCC method (unground costal cartilage method) and for methanol (determined using the UCC method) and ethanol (*p* = 0.058) concentration determined using the GCC method (ground costal cartilage method). Comparing the blood/cartilage ratios for ethanol alone, we found significantly lower ratio values for samples prepared using the GCC method (*p* < 0.001) (Table 3, Figure 4).

## 4. Discussion

The presented results contribute to the current knowledge of methanol distribution in the human body. A high positive correlation between methanol concentration in all types of studied cartilage and methanol concentration in the blood showed that this type of tissue could serve as an alternative material. The results for formic acid, a methanol metabolite, showed that it does not meet the requirements for its presence determination in cartilage tissues using the applied methods.

Earlier studies [25] showed a statistically significant, strong positive correlation between ethanol concentration in the blood and in cartilage (r = 0.925, *p* < 0.001) prepared according to the GCC method (ground costal cartilage method). The presented study shows that methanol concentration in the blood also strongly correlates with its concentration in costal cartilage (r = 0.8714, *p* < 0.001), even though the presented study used the UCC method (unground costal cartilage method), since we found no significant differences between the Pearson’s’ correlation coefficients mentioned above (*p* = 0.355). Comparing the obtained results with the previous studies [25], we may conclude that, within the UCC method, both methanol and ethanol show similar tissue “permeability”.

So far, few studies about mass methanol poisonings have analyzed the results of postmortem studies [30,33]. The ingestion of the same dose of methanol may result in different clinical symptoms. Therefore, a combination of multiple diagnosis methods may contribute to the forensic diagnosis of methanol poisoning more precisely, and the choice of the diagnostic method should be considered on an individual basis [27]. Using a wide range of alternative materials may help interpret the results and give medical–legal opinions. The high correlation coefficients between the blood and cartilage tissues for methanol suggest that methanol concentration in cartilage can be determined, and methanol behaves similarly to urine. Since the obtained results add another perspective to the distribution of xenobiotics in cartilage tissues, they may also be important in the context of using this type of tissue for regenerative medicine [34] or plastic surgery purposes [35].

## 5. Conclusions

Methanol presence in costal cartilage, epiglottis, and intervertebral disc cartilage was confirmed for the first time postmortem. Methanol concentration in all types of cartilage appositively correlated with this in femoral blood. Formic acid, a methanol metabolite, does not meet the requirements for its presence determination in cartilage tissues.

## Figures and Tables

**Table 1:** Descriptive statistics of quantitative variables analyzed in the victims of fatal methanol poisoning (*n* = 17).

Variable	*n*	Mean	SD	Median	Q_1_	Q_3_	x_min_	x_max_
Age	17	50.8	12.1	49.0	43.0	61.0	33.0	74.0
t1 [days]	15	5.8	4.9	3.0	1.0	12.0	1.0	13.0 ^#^
t2 [days]	17	7.2	3.4	7.0	6.0	8.0	1.0	16.0
Methanol [mg/mL or mg/g *]								
Blood	17	2.53	1.89	2.69	0.00	4.28	<0.1	5.14
Urine	16	3.42	2.58	3.38	1.10	5.63	<0.1	8.20
Costal cartilage *	17	0.66	0.53	0.67	0.00	1.12	<0.1	1.48
Epiglottis *	17	0.95	0.91	0.71	0.00	1.62	<0.1	3.16
Annulus fibrosis of intervertebral discs *	17	1.21	1.09	1.03	<0.1	2.04	<0.1	3.00
Formic acid [mg/mL or mg/g *]								
Blood	17	0.76	0.54	0.95	0.03	1.16	<0.01	1.49
Urine	15	2.81	2.69	2.95	0.04	4.62	<0.01	8.36
Costal cartilage *	17	0.14	0.17	0.07	0.01	0.20	<0.01	0.67
Epiglottis *	12	0.26	0.40	0.09	0.01	0.41	<0.01	1.38
Annulus fibrosis of intervertebral discs *	17	0.60	1.03	0.13	0.04	0.56	0.01	4.14

Legend: *—substance tissue concentration expressed in mg/g, t1—time from the last consumption of an industrial alcohol to death (#—t1 = 13 days is the time from the last consumption of and industrial alcohol to the corpse reveal), t2—time from death to autopsy, SD—standard deviation, Q_1_—lower quartile, Q_3_—upper quartile.

**Table 2:** Analysis of univariate linear regression for methanol concentration in blood and cartilage tissues of fatal methanol poisoning victims.

Concentration in the Blood	Concentration in Other Fluids or Tissues	β	SE (β)	r	*p*
Methanol	Urine	1.3043	0.1342	0.9290	<0.001
	Costal cartilage	0.2440	0.0355	0.8714	<0.001
	Epiglottis	0.3989	0.0713	0.8224	<0.001
	Annulus fibrosis of intervertebral discs	0.5137	0.0672	0.8920	<0.001
Formic acid	Urine	3.9346	0.8651	0.7836	<0.001
	Costal cartilage *	0.4381	0.3123	0.3405	0.181
	Epiglottis *	0.8091	0.3271	0.5382	<0.05
	Annulus fibrosis of intervertebral discs *	0.5762	0.3829	0.4696	0.171

Legend: β—regression coefficient, SE (β)—standard error for the regression coefficient, r—Pearson’s linear correlation coefficient, *—logarithmic transformation.

**Table 3:** Comparison of blood/urine and blood/cartilage concentration ratios for methanol and ethanol poisoning. Data for costal cartilage ethanol concentrations were taken from Tomsia et al. [25] study. Data are presented as medians (lower quartile;upper quartile).

Concentration Ratio	Methanol	Ethanol		*p*
Blood/urine	0.73 (0.72; 0.84)	0.79 (0.67; 1.00)		0.694
	UCC	UCC	GCC	
Blood/cartilage	3.69 (3.02; 4.92)	4.39 (3.10; 5.65)	2.53 (2.18; 3.35)	<0.001

Legend: GCC—costal cartilage prepared with the ground costal cartilage method [25], UCC—costal cartilage prepared with the unground costal cartilage method [25].

## Data Availability

Not applicable.
